# Interaction between Thiamethoxam and Deformed Wing Virus Type A on Wing Characteristics and Expression of Immune and Apoptosis Genes in *Apis mellifera*

**DOI:** 10.3390/insects13060515

**Published:** 2022-05-31

**Authors:** Patcharin Phokasem, Wannapha Mookhploy, Sasiprapa Krongdang, Chainarong Sinpoo, Panuwan Chantawannakul

**Affiliations:** 1 Bee Protection Laboratory, Department of Biology, Faculty of Science, Chiang Mai University, Chiang Mai 50200, Thailand; wannapha.mookhploy@gmail.com (W.M.); chainarong.sp@gmail.com (C.S.); 2 Faculty of Science and Social Sciences, Burapha University Sa Kaeo Campus, Sa Kaeo 27160, Thailand; sasiprapa.kr@buu.ac.th; 3 Environmental Science Research Center, Faculty of Science, Chiang Mai University, Chiang Mai 50200, Thailand

**Keywords:** apoptosis, DWV-A, immune, pathogens, pesticides, thiamethoxam

## Abstract

**Simple Summary:**

Honey bees are key pollinators in agricultural crops. Today, honey bee colonies in decline are a global concern as a result of various stressors, including pesticides, pathogens, honey bee health, and parasites. A healthy honey bee colony refers to colonies that are not exposed to biotic and abiotic stressors. In this study, we examine how thiamethoxam (pesticide) and deformed wing virus type A (DWV-A) interact in effects on honey bee health. The results revealed that the honey bees were infected with DWV-A and were additionally exposed to thiamethoxam, showing effects that increased the mortality rate, and crippled wings in newly emerged adult honey bees. Moreover, the exposure to thiamethoxam and DWV-A injection resulted in induced expression of immune genes (*hymenoptaecin* gene) while downregulation of two apoptosis genes (*caspase8-like*, *caspase9-like* genes). The impact interaction of pesticide and DWV-A have on the expression of apoptosis genes can directly affect viral susceptibility in the honey bee host.

**Abstract:**

Honey bees are economically important insects for crop pollination. They play a significant role as pollinators of wild plants and agricultural crops and produce economical products, such as honey, royal jelly, wax, pollen, propolis, and venom. Despite their ecological and economical importance, the global honey bee population is in decline due to factors including pathogens, parasites, intensive agriculture, and pesticides. Moreover, these factors may be interlinked and exacerbate the loss of honey bees. This study aimed to investigate the interaction between a pesticide, thiamethoxam, and deformed wing virus type A (DWV-A) to honey bees and the effects on survival rate, wing characteristics, and expression of immune and apoptosis genes in *Apis mellifera*. We described the potential interaction between thiamethoxam and DWV-A on honey bee wing characteristics, DWV-A loads, and the expressions of immune (*defensin*, *abaecin*, and *hymenoptaecin*) and apoptosis genes (*buffy*, *apaf1*, *caspase3-like*, *caspase8-like*, and *caspase9-like*). Honey bee larvae were fed with three different thiamethoxam doses (0.001, 1.4, and 14.3 ng/µL of the diet). Then, thiamethoxam-treated white-eyed pupae were injected with 10^7^ copy numbers/honey bee of the DWV-A genome. The interaction between thiamethoxam and DWV-A caused a high mortality rate, crippled wings in newly emerged adult honey bees (100%), and resulted in induced expression of *hymenoptaecin* gene compared to the control group, while downregulation of *caspase8-like*, *caspase9-like* genes compared to the DWV injection group. Therefore, the potential interaction between thiamethoxam and DWV-A might have a deleterious effect on honey bee lifespan. The results from this study could be used as a tool to combat DWV-A infection and mitigate pesticide usage to alleviate the decrease in the honey bee population.

## 1. Introduction

The western honey bee, *Apis mellifera*, is the main pollinator of wild plants and agricultural crops. They also produce honey and other hive products such as royal jelly, wax, pollen, propolis, and venom [1,2,3]. The global and rapid loss of honey bee colonies has been associated with various factors, including pesticides, pathogens, honey bee health, and parasites [4]. However, these losses are thought to be largely attributed to the pesticide as well as emergent pathogens, including viruses [5]. Furthermore, viruses and pesticides can be concurrent threats to honey bee colonies, as honey bees infected with different pathogens encounter pesticides when collecting pollen and nectar [6,7].

When honey bees forage pollen, pesticide residue in crops up to 10 km away can pollute collected pollen and nectar and consequently cause pesticide contamination in colonies. Thiamethoxam (nitro-substituted neonicotinoid) is now the most commonly used insecticide in crops worldwide for seed coatings or directly sprayed on crops [8,9]. Previous studies have shown that thiamethoxam had negative effects on honey bees in the larval stage [10,11,12,13], pupal stage [14], and adult stage [15,16,17,18]. Moreover, neonicotinoid insecticides are likely to cause changes in honey bee physiology, such as hypopharyngeal gland development [19,20], honey bee behavior [21,22,23], colony development [24], foraging [25,26], and memory and learning [27,28,29].

Among honey bee pathogens, viruses have been one of the main culprits associated with honey bees’ colony decline [30,31]. To date, about 26 honey bee viruses have been described, most of which are single-stranded RNA viruses, primarily belonging to the Dicistroviridae and Iflaviridae families [30]. The most common honey bee viruses include acute bee paralysis virus (ABPV), black queen cell virus (BQCV), chronic bee paralysis virus (CBPV), deformed wing virus (DWV), Israeli acute bee paralysis virus (IABPV), Kashmir bee virus (KBV), and sacbrood virus (SBV) were detected in honey bee colonies [5,32]. DWV is widespread and dominant in *A. mellifera*, positively correlated with varroa mites and tropilaelaps mites infestation [32,33,34,35]. DWV causes crippled wings and reduced body size in adult honey bees [36]. Several studies have documented that DWV has been linked to colony losses [32,37,38]. Three master variants of DWV (DWV-A, DWV-B, and DWV-C) have been discovered, with DWV-A being the most widespread variant [39,40,41]

Effects of co-exposure between pesticides and honey bee viruses have already been reported, resulting in an increase in DWV loads [42], BQCV loads [43], and CBPV loads [44] in honey bees. The effect of these factors has also been found to cause higher mortality rates in honey bee larvae [45]. Moreover, the change in gene expression pattern has been observed in immune and detoxification genes in honey bees [44]. Although the co-exposure of honey bees to DWV and thiamethoxam were investigated in previous studies [45], information on the relationship between crippled wings honey bees and gene pattern is still scarce. In this study, we described the effects of DWV-A infection and different concentrations of thiamethoxam treatment on the survival, viral loads, wing characteristics, and expressions of immune and apoptosis genes in newly emerged adult honey bees.

## 2. Materials and Methods

### 2.1. Honey Bee Samples

Seven *Apis mellifera* colonies maintained at Bee Protection Laboratory (BeeP) apiary, Chiang Mai University, Thailand (18°48′14.3″ N 98°57′22.2″ E) during 2018–2019 were used in this study. The crippled honey bees were collected from four colonies kept without ectoparasitic mites treatment. Three honey bee colonies, no visible clinical symptoms, and low/no ectoparasitic mites infestation were used for in vitro larval rearing.

### 2.2. Preparation of Deformed Wing Virus Type A (DWV-A) Lysate

The crippled adult honey bees were collected from *A. mellifera* colonies to prepare a DWV-A lysate. Five crippled adult honey bees were frozen in liquid nitrogen and crushed with a mortar. The ground crippled adult honey bees were suspended in 5 mL of phosphate buffer solution (pH 7.4) and then centrifuged at 6440× *g* for 10 min at 4 °C (K3 Series, Centurion Scientific Ltd., London, UK). The supernatant was collected after centrifugation and filtered through a 0.2-micron filter (Millipore, Merck, Darmstadt, Germany) to eliminate bacteria, fungi, and Nosema. The absence of six common honey bee viruses (acute bee paralysis virus (ABPV) [46], black queen cell virus (BQCV) [46], chronic bee paralysis virus (CBPV) [46], Israeli acute bee paralysis virus (IABPV) [47], Kashmir bee virus (KBV) [46], and sacbrood virus (SBV) [46]) in the lysate was confirmed by quantitative real-time PCR (qRT-PCR). The sequence of primers used is shown in Table S1. Lysate without all six common honey bee viruses was used for this study. The lysate was kept at −80 °C until use [48]. The level of DWV genome equivalents in the lysate was measured using the same qRT-PCR technique described later in the Materials and Methods (Section 2.6 and Section 2.7).

### 2.3. Diet and Larval Feeding

The first instar larval stage of *A. mellifera* was grafted onto an artificial diet plate. The levels of different sugar and yeast extract concentrations in food were provided for each developmental larval stage to meet the nutritional requirements. The artificial diet consisting of 50% *w*/*w* of royal jelly and 50% *w*/*w* of distilled water that contained either diet A (12% *w*/*v* glucose, 12% *w*/*v* fructose, and 2% *w*/*v* yeast extract), or diet B (15% *w*/*v* glucose, 15% *w*/*v* fructose, and 3% *w*/*v* yeast extract), or diet C (18% *w/v* glucose, 18% *w*/*v* fructose, and 4% *w*/*v* yeast extract) was refreshed every day. On the first and second days of in vitro rearing, each larva was fed with diet A, and then diet B was fed on the third day. Finally, diet C was fed on the fourth, fifth, and sixth days of the larvae developmental stage. Plates of larvae were incubated at 34 ± 1 °C and 96% RH [49].

### 2.4. Exposure to Thiamethoxam

Thiamethoxam was mixed in with diet C at three concentrations, including 0.001 (LT group), 1.4 (MT group), and 14.3 (HT group) ng/µL of the diet (note that the concentration of 0.001 ng/µL was the equivalent level of residues found in nectar, pollen, and beebread) [13]. The medium and high concentrations of thiamethoxam were selected according to a previous study [12], which were the lethal and sub-lethal concentrations of thiamethoxam to honey bee larvae reared in vitro. Diet C with no thiamethoxam was used in the control group (C group). The experimental groups were fed with diet C at different concentrations on the 4th day after grafting. After that, larvae received only food without the insecticide on the 5th and 6th days. On the 4th, 5th, and 6th days, each larva was fed 30, 40, and 50 ng/µL of diet C, respectively [12]. Overall, 105 honey bee larvae were subject to each treatment. Larval mortality was checked individually by observation under a stereomicroscope (Olympus, Tokyo, Japan) until they developed into the white-eyed pupae stage.

### 2.5. Injection of DWV-A to Honey Bee White-Eyed Pupae

The white-eyed pupae were collected and divided into 9 groups. Thiamethoxam-treated white-eyed pupae were injected laterally between the second and third tergite of the abdomen with 2 µL per honey bee of PBS containing 10^7^ copy numbers/honey bee of DWV-A genome. Thiamethoxam-treated white-eyed pupae were divided into six groups: LT/V- (treated with 0.001 ng/µL thiamethoxam); LT/NC (treated with 0.001 ng/µL thiamethoxam with PBS injection); LT/V+ (treated 0.001 ng/µL thiamethoxam with DWV-A injection); MT/V- (treated 1.4 ng/µL thiamethoxam); MT/NC (treated 1.4 ng/µL thiamethoxam with PBS injection); MT/V+ (treated 1.4 ng/µL thiamethoxam with DWV-A injection). Thiamethoxam-untreated white-eyed pupae were injected with 2 µL per honey bee of 10^7^ copy numbers/honey bee of DWV-A genome as a positive DWV-A control group (PC group). White-eyed pupae that were not treated with thiamethoxam and PBS injected were used as a negative control group (NC group). The PBS injection treatments were used as a control for the injection [45,50]. White-eyed pupae that were not treated with thiamethoxam and not injected were used as a handling control group (C group). All white-eyed pupae were incubated at 34 ± 1 °C and 70% RH until developing into newly emerged adult honey bees [51,52]. The honey bee survival rate was monitored during development.

### 2.6. RNA Extraction and cDNA Synthesis

Total RNA of adult honey bees was individually extracted by using TRIzol^®^ (Invitrogen, Carlsbad, CA, USA) following the manufacturer’s protocol. RNA concentration and quantity were determined using a BioDrop Duo spectrophotometer (BioDrop Ltd., Cambridge, UK). Reverse transcription was performed from 1 μg RNA to cDNA using the Tetro cDNA synthesis kit (Bioline, Alexandria, NSW, Australia) following the manufacturer’s protocol.

### 2.7. Quantitative Real-Time PCR Parameters

The number of DWV-A genome copies was determined by the absolute quantification method. The standard curve was established by plotting seven 10-fold dilutions of DWV-A insert in TOPO ^®^TA Cloning^®^ plasmid (Invitrogen, Carlsbad, CA, USA). The qRT-PCR was performed on BioRad iQ^TM^ 5 (Bio-Rad Crop., Hercules, CA, USA), using SensiFAST SYBRR^®^ No-ROX Kit master mix (Bioline, Alexandria, NSW, Australia). The amplification was performed in a 20 μL reaction volume using SensiFAST SYBR^®^ No-ROX Mix, consisting of 10 µL of 2x SensiFAST SYBR^®^ No-ROX Mix, 0.8 µL of each 10 µM primer, 1 μL of 10-fold diluted cDNA and nuclease-free water to adjust the volume to 20 μL. For amplification step with the following profile was used: 50 °C for 30 min and 95 °C for 10 min, followed by 40 cycles of 95 °C for 30 s, 55 °C for 1 min, and 72 °C for 30 s. The melting curve was generated from 55 °C to 95 °C in 0.5 °C/s increments. The sequence of DWV-A [53], and housekeeping genes [54,55] primers is described in Table S1. 

Relative quantification in real-time PCR was determined in antimicrobial peptides (AMPs), and apoptosis-related genes [50,56]. Ribosomal protein subunit 5 (RPS5) and *β*-actin were used as housekeeping genes for all primers shown in Table S1. qRT-PCR was performed as described above. All reactions were carried out using a thermal program of 95 °C for 30 s followed by 40 cycles of 95 °C for 30 s, 60 °C for 30 s, and 72 °C for 1 min. The final qRT-PCR amplification was confirmed by the analysis of the melting curve generated from 55 °C to 95 °C in 0.5 °C/s increments. Each experiment was performed in triplicate, and negative controls (no template) were included in each reaction. Gene expression was calculated as 2^−ΔΔCT^ [57].

### 2.8. Statistical Analysis

The survival of white-eyed pupae and newly emerged adult honey bees were established using Kaplan–Meier survival statistics with the log-rank test. Log-transformed DWV-A loads and gene transcripts were analyzed using one-way ANOVA (Welch ANOVA in cases of unequal variance) followed by the Games-Howell post-hoc *t*-test. The data were analyzed using generalized linear models (GLMs) to evaluate significant variations among treatments and genes, with treatments and genes as fixed factors, and the interaction was included. *p*-values less than 0.05 were noted as significant. All statistical analyses were tested using the SPSS v 25 program (IBM Corp., Armonk, NY, USA). 

## 3. Results

### 3.1. Effects of Thiamethoxam on Survival of Larvae to White-Eyed Pupae

**Figure 1:**
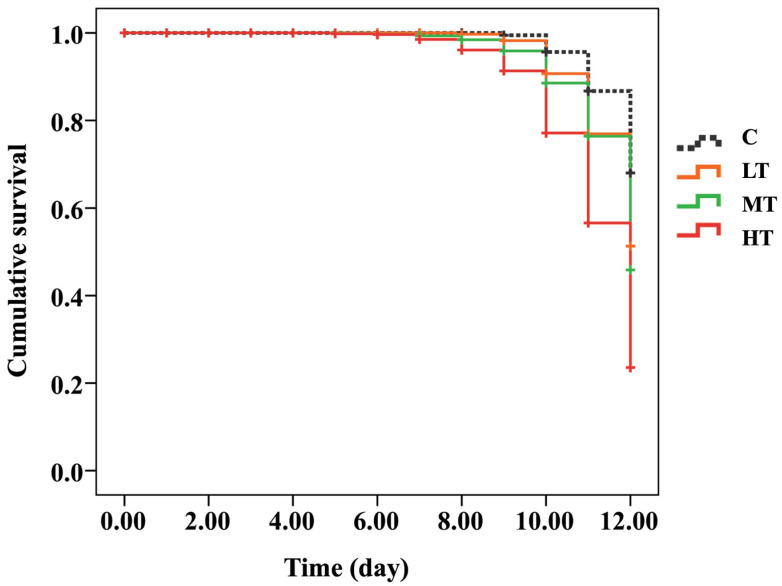
Kaplan–Meier survival curve of white-eyed pupae that were treated with three concentrations of thiamethoxam (0.001, 1.4, and 14.3 ng/µL) and control (untreated thiamethoxam) in the larval stage.

The cumulative survival curves of *A. mellifera* white-eyed pupae were significantly different between the C group (thiamethoxam-untreated) and thiamethoxam-treated groups after 12 days post feed (Kaplan–Meier log-rank test, x^2^ = 170.826, *p* < 0.0001; Figure 1 and Figure S1). The survival rate of the C group (90%) was not significantly different compared to the LT group (70%) (log-rank test, *p* = 0.059). In addition, the survival rates between MT (39%) and HT (22%) groups were not significantly different (log-rank test, *p* = 0.128; Figure 1 and Figure S1 and Table S2). Honey bees fed with the highest thiamethoxam dose (14.3 ng/µL; HT group) showed a significantly lower survival rate than the C group (log-rank test, *p* < 0.0001), and honey bees fed with 0.001 (LT group), 1.4 (MT group) ng/µL of thiamethoxam (log-rank test, *p* = 0.013 and 0.028, respectively) (Figure 1 and Table S2).

### 3.2. Effects of Co-Exposure of Thiamethoxam and DWV-A on the Survival of White-Eyed Pupae to Newly Emerged Adult Honey Bees

**Figure 2:**
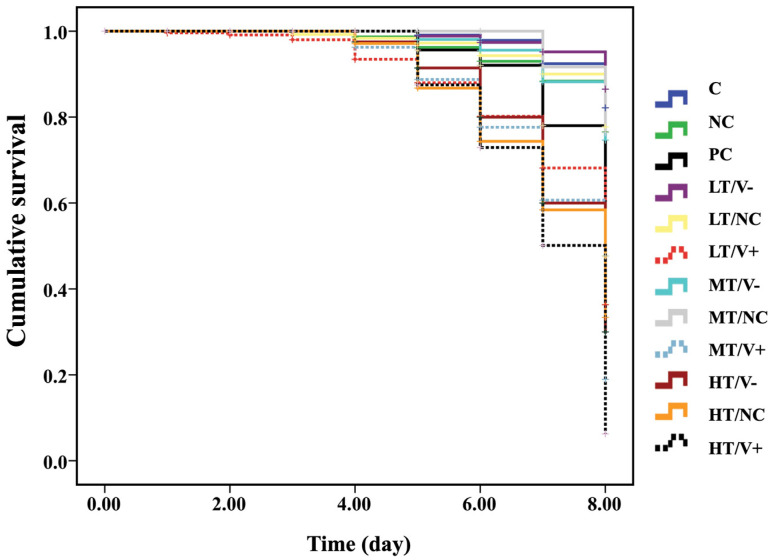
Kaplan–Meier survival curve of newly emerged adult honey bees treated with three concentrations of thiamethoxam (0.001, 1.4, and 14.3 ng/µL) in the larval stage that were injected with DWV-A, PBS, and control (not treated with thiamethoxam and uninfected group) in the white-eyed pupal stage.

The cumulative survival curves of *A. mellifera* newly emerged adult honey bees were significantly different between the control and treated groups 8 days post injection (Kaplan–Meier log-rank test, x^2^ = 131.182, *p* < 0.0001; Figure 2). There was no significant difference in cumulative survival rates among the C group (thiamethoxam-untreated with no DWV-A injection) (89%), NC (thiamethoxam-untreated with PBS injection) (87%), LT/V- (91%), LT/NC (86%), MT/V- (85%), and MT/NC (83%) (log-rank test, *p* > 0.05). However, these treatment groups showed higher cumulative survival rates when compared to groups injected with DWV-A. The injection with DWV-A-untreated thiamethoxam group (PC group) and LT/V+, MT/V+, and HT/V+ groups showed survival rates of 61%, 47%, 50%, and 13%, respectively. The HT/V- and HT/NC groups resulted in survival rates of 13% and 14%, respectively (at *p* < 0.05, Figure 2 and Figure S2, and Table S3). Moreover, PC groups showed higher cumulative survival rate than LT/V+, MT/V+, and HT/V+ groups at *p*-value = 0.012, <0.0001, and <0.0001, respectively (Figure 2). A significant effect of interaction between thiamethoxam and DWV-A on mortality was found in all co-exposure groups when compared with DWV-A alone or thiamethoxam alone, except for the high dose thiamethoxam groups (HT/V- and HT/NC).

### 3.3. Effects of Co-Exposure of Thiamethoxam and DWV-A on Wing Characteristics of Newly Emerged Adult Honey Bees

**Figure 3:**
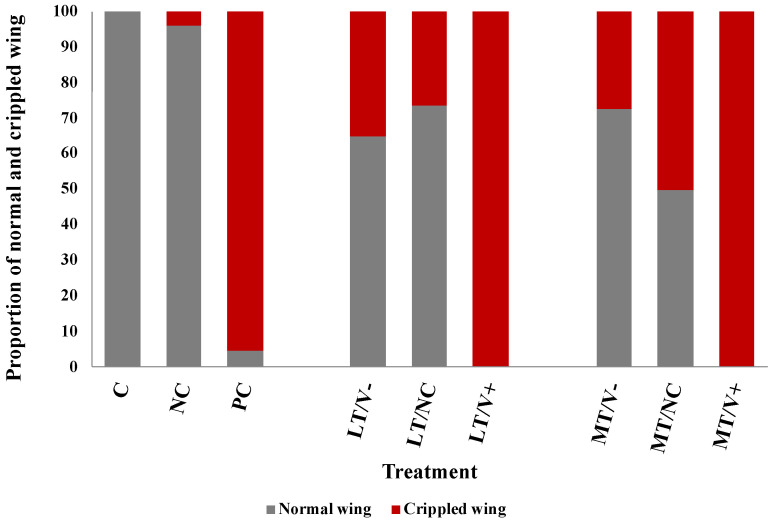
Percentages of the normal and crippled wings of newly emerged adult honey bees after being treated with thiamethoxam at 0.001 and 1.4 ng/µL in the larval stage and injected with DWV-A and PBS in the white-eyed pupal stage. The untreated and uninjected larvae were used as controls.

All newly emerged adult honey bees showed normal wings in the C group (100%) and the NC group (96%). Newly emerged adult honey bees that were not treated with thiamethoxam and injected with DWV-A (PC group) showed both normal and deformed wings at 5% and 95%, respectively. All thiamethoxam-treated groups were investigated for the crippled wings. The groups that were subject to 0.001 ng/µL of thiamethoxam (LT/V- group) and 0.001 ng/µL of thiamethoxam with PBS (LT/NC group) showed normal wing at 65% and 74%, respectively. The groups that were subject to 1.4 ng/µL of thiamethoxam (MT/V- group) and 1.4 ng/µL of thiamethoxam with PBS (MT/NC group) showed normal wing at 73% and 50%, respectively. The results showed that all concentrations of thiamethoxam treatments that were injected with DWV-A resulted in crippled wings in newly emerged adult honey bees (100%) (Figure 3). The survival rate of the HT/V+, HT/V-, and HT/NC groups was very low, and, therefore, the wing characteristic analysis was not performed.

### 3.4. DWV-A Loads in Newly Emerged Adult Honey Bees

**Figure 4:**
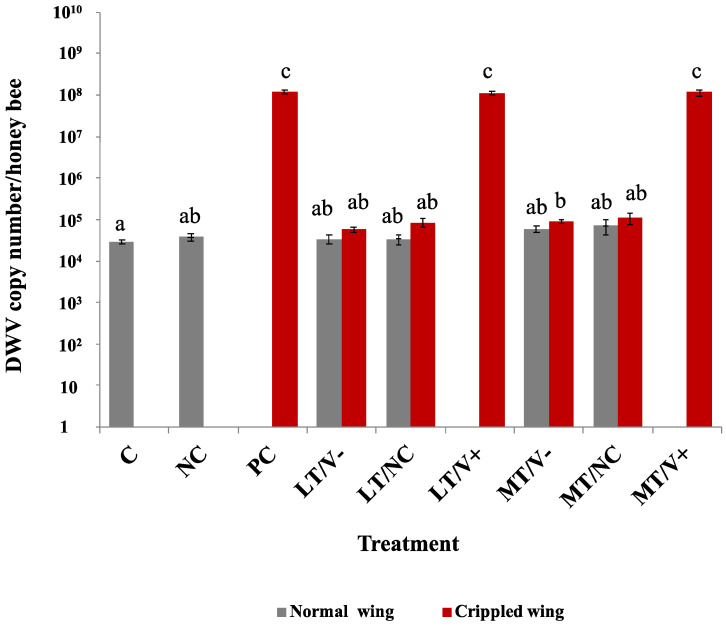
DWV-A loads in newly emerged adult honey bees treated with thiamethoxam at 0.001 and 1.4 ng/µL in the larval stage and injected with DWV-A and PBS in the white-eyed pupal stage. The control group was not treated with thiamethoxam and not injected. Vertical bars represent means ± SEM. One-way ANOVA with Games–Howell post-hoc test was used. The lowercase letters indicate significant differences at *p*-values less than 0.05.

Low DWV-A loads were detected in C and NC groups in newly emerged adult honey bees (2.9 × 10^4^ ± 8.0 × 10^3^ and 3.8 × 10^4^ ± 1.6 × 10^4^ copy numbers/honey bee, respectively). The DWV-A levels of the MT/V- group showed a statistically significant difference in DWV-A levels compared to the C group (*p* = 0.031). The PC groups had higher DWV-A levels compared to the C group and all treatment groups at a *p*-value less than 0.05, except LT/V+ and MT/V+ groups (Figure 4 and Table S4). Crippled wings honey bees in the PC, LT/V+, and MT/V+ groups showed DWV-A loads of 1.2 × 10^8^ ± 1.4 × 10^7^, 1.1 × 10^8^ ± 1.1 × 10^7^, and 1.2 × 10^8^ ± 1.9 × 10^7^ copy numbers/honey bee, respectively, and there was no statistically significant difference in DWV-A loads between each other (*p* > 0.05). The interaction between thiamethoxam and DWV-A did not result in a significant modulation of DWV-A loads.

### 3.5. Immune- and Apoptosis-Related Gene Expression in Newly Emerged Adult Honey Bees

**Figure 5:**
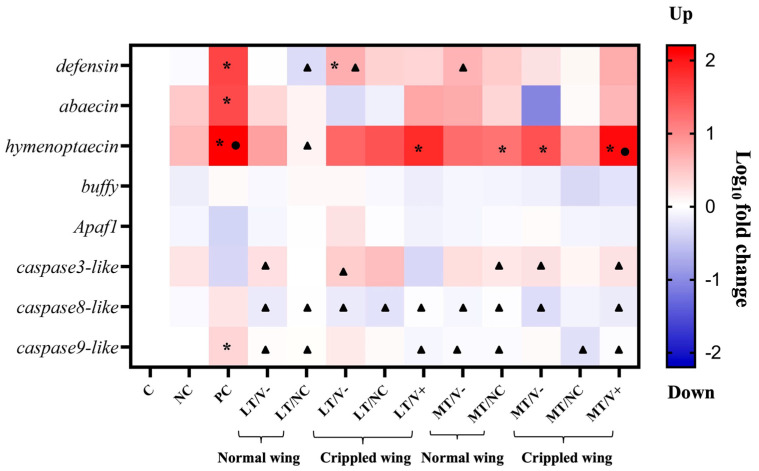
Heatmap of immune and apoptosis genes expression levels in newly emerged adult honey bees. A black asterisk (*) indicates a significant difference between the treatment compared to the control group. A black circle (●) indicates a significant difference between the treatment compared to the PBS group. A black triangle (▲) indicates a significant difference between the treatment compared to the DWV-A group (*p* < 0.05; Welch ANOVA and Games-Howell).

White-eyed honey bee pupae treated **with** 0.001 and 1.4 ng/µL thiamethoxam and control (thiamethoxam-untreated) in the larval stage were injected with PBS and DWV-A. Three immune (*defensin*, *abaecin*, and *hymenoptaecin*) and five apoptosis-related genes (*buffy*, *apaf1*, *caspase3-like*, *caspase8-like*, and *caspase9-like*) were investigated at the newly emerged adult honey bee stage. The results showed no significant differences in the expressions of *buffy* and *apaf1* (Welch ANOVA, *p* = 0.062 and 0.095, respectively) in all experimental groups. In contrast, there were statistically significant differences in the expressions of six genes, including *defensin*, *abaecin*, *hymenoptaecin*, *caspase3-like*, *caspase8-like*, and *caspase9-like* (Welch ANOVA, all genes, *p* < 0.01). PC group showed an upregulation in three immune genes, including *defensin*, *abaecin*, and *hymenoptaecin,* compared to the C group (Games-Howell, *p* = 0.003, 0.034, and 0.011, respectively) (Figure 5 and Tables S5–S7). The expressions of two immune genes (*defensin* and *abaecin*) were lower in all groups treated with thiamethoxam and DWV-A injection than in the PC group, though not significantly different. Only the *hymenoptaecin* gene showed slightly higher upregulation in LT/V+ and MT/V+ groups than in the C group (Games–Howell, *p* = 0.005 and 0.006, respectively) (Figure 5 and Table S7). Honey bees that were treated with 1.4 ng/µL thiamethoxam and with DWV-A injection (MT/V+ group) showed an upregulation of the *caspase3-like* gene (Games-Howell, *p* = 0.008) compared to the PC group (Figure 5 and Table S8). The *caspase8-like* and *caspase9-like* genes showed the highest upregulation in the PC group, but only *caspase9-like* was significantly different compared to the control at *p* = 0.018 (Figure 5 and Tables S9 and S10). Moreover, the mRNA levels of the two genes were significantly suppressed in LT/V+ and MT/V+ groups compared to the PC group (Games-Howell, *caspase8-like p* = 0.046 and 0.031, respectively, and *caspase9-like p* = 0.014 and 0.026, respectively).

Gene expression was significantly influenced by treatments and genes, and the interaction was also significant (GLMs: *p* < 0.001 for treatments; *p* < 0.001 for genes; and *p* < 0.001 for the interaction).

## 4. Discussion

Our study provides an insight into the effects on survival, DWV-A loads, wing characteristic, and expression of immune and apoptosis genes of *Apis mellifera* after exposure to different doses of thiamethoxam and DWV-A infection in newly emerged adult honey bees. Our results are consistent with previous reports, as we found that honey bees exposed to thiamethoxam in the larval stage had a significantly reduced survival rate in the white-eyed pupal stage [13,39]. Moreover, the combined effect of thiamethoxam and DWV-A further decreased the survival rate of newly emerged adult honey bees. Coulon et al. [45] also reported that a high dosage of thiamethoxam decreased the survival rate of honey bees after being injected with DWV. In this study, we showed that treatment with a low concentration of thiamethoxam (environmental dose in the colony) induces increased crippled wings in newly emerged adult honey bees. Nevertheless, there are limitations in this study that could be addressed in future research. The study used two high concentrations (1.4 and 14.3 ng/µL) that are not environmentally relevant. These concentrations not only induced high mortality but also resulted in an uneven number of individual tested.

Previous studies have shown that thiamethoxam caused changes in honey bee physiology [19,20]. Honey bees exposed to pesticides in the larval stage developed deformed physical characteristics in the adult stage, such as wing malformation, stunted bodies, and crippled legs [58]. Our study demonstrated that the effects of treatment with only thiamethoxam induce increased wing deformity in newly emerged adult honey bees. Moreover, numerous studies have also demonstrated that DWV is also the cause of crippled wings in honey bees [56,59]. Our result showed that honey bees exposed to thiamethoxam and DWV-A stressors had a high percentage of crippled wings as newly emerged adult honey bees. As a consequence, exposure to pesticides and DWV-A in honey bee colonies may impact the ability of adult honey bees to perform duties and forage effectively, leading to a decreased rate of colony survival. 

In the present study, DWV-A levels of honey bees co-exposed to DWV-A and thiamethoxam were significantly higher than in treated groups, except for the PC group. Thus, the combination of neonicotinoid insecticides and DWV infection induced significantly higher DWV viral loads in honey bees [42]. These results coincide with the low survival rate of honey bees co-exposed to DWV-A and thiamethoxam, suggesting the effect between thiamethoxam and DWV-A infection on honey bee survival. 

The immune-related gene expressions of honey bees co-exposed to thiamethoxam and DWV-A were upregulated in newly emerged adult honey bees. However, only the *hymenoptaecin* gene was significantly upregulated compared to the control group. The *hymenoptaecin* gene is one of the antimicrobial peptides that have been identified in honey bees to be active against microorganisms [60]. AMPs play a crucial role in the insect immune system and contribute to individual and social immunity [50,61,62]. Previous studies have indicated that the expressions of AMP genes in honey bees were upregulated after the invasion of pathogens, including microsporidian *Nosema* [63], *Paenibacillus larvae* [64], viruses [56], and ectoparasitic mites [65]. Viral infection within the host via viral entry, replication, and spreading can induce the antiviral innate immune responses [66]. Upregulation of several AMP genes, including *abaecin*, *hymenoptaecin*, and *defensin*, was also shown in other studies where honey bees were infected with DWV-A [56]. Treatment with thiamethoxam led to the downregulation of *abaecin* and *defensin* genes in crippled wings adult honey bees. Interestingly, honey bees exposed to thiamethoxam and DWV-A injection were also found to downregulate *abaecin* and *defensin* genes, implying immunological toxicity.

We found that co-exposure to thiamethoxam and DWV-A decreased the expression of apoptosis-related genes and significantly down-regulated *caspases8-like* and *caspases9-like* genes. The *caspases* gene is known to be related to programmed cell death and is associated with the final proteases in apoptosis [67]. Apoptosis is an important component of various processes, including normal cell development, embryonic development, function of the immune system, hormone-dependent atrophy, and chemical-induced cell death [68,69]. Evidence from previous studies suggested that virus infection induced apoptosis in insects and that the infection was mitigated by the elimination of the infected cells [70,71,72]. Honey bees injected with DWV had suppressed the expression of caspases in the pupal stage, which likely promoted the virus survival in hosts [56]. Honey bee co-exposure groups showed a strong alteration of immune gene expressions and downregulation of two apoptosis genes. Further studies are needed to investigate in greater detail the mechanisms for the viruses and pesticides that destroy immune pathways and the ability of viral replication in honey bee hosts. 

## 5. Conclusions

This study showed the combined effect of DWV-A and thiamethoxam on *A. mellifera*, resulting in an increased mortality rate, crippled wings, and increased DWV-A loads. Our finding showed that honey bees exposed to thiamethoxam and DWV-A could intensify DWV-A infection, which could result in long-term physical deformity and decreased honey bees’ life span. Data from our investigation revealed that gene expression patterns changed in each treatment group. The effect of both thiamethoxam and DWV-A results in the transcriptome imbalance, which may also have an effect on stress recovery and, subsequently, on honey bees’ survival rate. Therefore, the results of our study could be explained by a negative interaction between thiamethoxam and DWV-A on honey bee lifespan in laboratory conditions. Future studies should be undertaken to examine the effects of pesticide exposure and viral infection occurring under field conditions.

## Data Availability

Data is contained within the article and supplementary material.

## Supplementary Materials

The following supporting information can be downloaded at: https://www.mdpi.com/article/10.3390/insects13060515/s1, Table S1: Primers used for qRT-PCR amplification in this study, Table S2: Statistic of survival in white-eyed pupae honey bees, Table S3: Statistic of survival in newly emerged adult honey bees, Table S4: Statistic of DWV-A load in newly emerged adult honey bees, Table S5: Statistic of *defensin* in newly emerged adult honey bees, Table S6: Statistic of *abaecin* in newly emerged adult honey bees, Table S7: Statistic of *hymenoptaecin* in newly emerged adult honey bees, Table S8: Statistic of *caspase3*-like in newly emerged adult honey bees, Table S9: Statistic of *caspase8*-like in newly emerged adult honey bees, Table S10: Statistic of *caspase9*-like in newly emerged adult honey bees, Figure S1: The percentage of survival rate of white-eyed pupae honey bees at 12 days post feed, Figure S2: The percentage of survival rates in newly emerged adult honey bees.
