# Supplementary material for: Interaction between Thiamethoxam and Deformed Wing Virus Type A on Wing Characteristics and Expression of Immune and Apoptosis Genes in Apis mellifera
Source: Insects. 2022 May 31;13(6):515. doi: 10.3390/insects13060515 (PMC9225052; doi:10.3390/insects13060515)
Supplement: Supplementary file 1 [file insects-13-00515-s001.zip › insects-1723019-supplementary.pdf]

Supplementary Materials

Title Interaction Between Thiamethoxam and Deformed Wing Virus type A on Wing Characteristics and Expression of Immune and Apoptosis Genes in *Apis mellifera*

Patcharin Phokasem, Wannapha Mookhploy, Sasiprapa Krongdang, Chainarong Sinpoo, and Panuwan Chantawannakul

Table S1 Primers used for qRT-PCR amplification in this study

Genes	Primer	Sequence 5'-3'	References
Housekeeping			
Ribosomal protein S5 (RPS5)	AmRPS5.F	AATTATTTGGTCGCTGGAATTG	[54]
	AmRPS5.R	TAACGTCCAGCAGAATGTGGTA	
β-Actin	Actin.F	TTGTATGCCAACACTGTCCTTT	[55]
	Actin.R	TGGCGCGATGATCTTAATTT	
Immune-related			
Antibacterial peptide abaecin	Abaecin.F	CAGCATTCGCATACGTACCA	[50]
	Abaecin.R	GACCAGGAAACGTTGGAAAC	
Antibacterial peptide defensin	Defensin.F	TGCGCTGCTAACTGTCTCAG	[50]
	Defensin.R	AATGGCACTTAACCGAAACG	
Antibacterial peptide hymenoptaecin	Hymenopt.F	CTCTTCTGTGCCGTTGCATA	[50]
	Hymenopt.R	GCGTCTCCTGTCATTCCATT	
Apoptosis-related			
Apoptotic peptidase activating factor 1-like	Apaf-1.F	ACAGATGATAATTTACAGGTGTGGG	[56]
	Apaf-1.R	TCCGTTCACTCTATCCGTTTGT	
Bcl-2 family proteins-like	Buffy.F	TGCCGATGCCTGAAAAGTCT	[56]
	Buffy.R	TCTGCGATAAGGTTGGCCTG	
Cysteine proteases3-like	Caspase3-like.F	CATGCACAGAAGAAATTCGCCA	[56]
	Caspase3-like.R	GTTCGTCCCGTTTCGTTGTG	
Cysteine proteases8-like	Caspase8-like.F	AAAACAATTGATGCAGTAGGGG	[56]
	Caspase8-like.R	TTCTGGAAATTGAAAATCGGAAGA	
Cysteine proteases9-like	Caspase9-like.F	TGGCCAAAGCTTGTTGAAAATCA	[56]
	Caspase9-like.R	ATGCAAAAGGTCCCCGTGTT	
	DWV.F	CGAAACCAACTTCTGAGGAA	[53]

Deformed wing virus	DWV.R	GTGTTGATCCCTGAGGCTTA	
Black queen cell virus	BQCV-qF7893	AGTGGCGGAGATGTATGC	[46]
	BQCV-qB8150	GGAGGTGAAGTGGCTATATC	
Kashmir bee virus	KBV-qF6639	CCATACCTGCTGATAACC	[46]
	KIABPV-qB6707	CTGAATAATACTGTGCGTATC	
Sacbrood virus	SBV-qF3164	TTGGAACTACGCATTCTCTG	[46]
	SBV-qB3461	GCTCTAACCTCGCATCAAC	
Acute bee paralysis virus	ABPV-qF6548	TCATACCTGCCGATCAAG	[46]
	KIABPV-qB6707	CTGAATAATACTGTGCGTATC	
Chronic bee paralysis virus	CBPV1-qF1818	CAA CCTGCCTCA ACACAG	[46]
	CBPV1-qB2077	AAT CTGGCAAGG TTGACTGG	
Israeli acute paralysis virus	IAPV.F	CCATGCCTGGCGATTCAC	[47]
	IAPV.R	CTGAATAATACTGTGCGTATC	

Table S2 Statistic of survival in white-eyed pupae honey bees

**Treatment**		**Log-rank test:X^2 and *p-value***			
		**control**	**Thaimethoxam**		
			**0.001 ng/µl**	**1.4 ng/µl**	**14.3 ng/µl**
**control**					
**Thiamethoxam**	**0.001 ng/µl**	0.059			
	**1.4 ng/µl**	<0.0001	0.013		
	**14.3 ng/µl**	<0.0001	0.028	0.128	

Table S3 Statistic of survival in newly emerged adult honey bees

**Treatment**		**Log-rank test:X^2 and *p value***											
		**control**			**Thiamethoxam**								
		**control**	**PBS**	**DWV**	**0.001 ng/µl**	**0.001ng/µl +PBS**	**0.001 ng/µl+ DWV**	**1.4 ng/µl**	**1.4 ng/µl +PBS**	**1.4 ng/µl DWV**	**14.3 ng/µl**	**14.3 ng/µl+ PBS**	**14.3 ng/µl+ DWV**
**control**	**control**												
	**PBS**	0.215											
	**DWV**	<0.001	0.014										
**Thiamethoxam**	**0.001 ng/µl**	0.633	0.110	<0.001									
	**0.001ng/µl +PBS**	0.351	0.811	0.015	0.189								
	**0.001 ng/µl +DWV**	<0.001	<0.001	0.012	<0.001	<0.001							
	**1.4 ng/µl**	0.394	0.865	0.049	0.222	0.976	0.001						
	**1.4 ng/µl+ PBS**	0.842	0.455	0.016	0.556	0.588	<0.001	0.599					
	**1.4 ng/µl+ DWV**	<0.001	<0.001	<0.001	<0.001	<0.001	0.310	<0.001	<0.001				
	**14.3 ng/µl**	<0.001	0.005	0.044	<0.001	<0.001	0.800	0.002	<0.001	0.622			
	**14.3 ng/µl +PBS**	<0.001	0.016	0.031	<0.001	<0.001	0.649	0.001	<0.001	0.792	0.863		
	**14.3 ng/µl +DWV**	<0.001	<0.001	<0.001	<0.001	<0.001	0.065	<0.001	<0.001	0.331	0.211	0.316	

Table S4 Statistic of DWV load in newly emerged adult honey bees

Games-Howell Post-Hoc Test															
			Normal wing						Deformed wing						
	Characteristic	Treatment	control	PBS	0.001 ng/µl	0.001ng/µl +PBS	1.4 ng/µl	1.4 ng/µl +PBS	DWV	0.001ng/µl	0.001 ng/µl +PBS	0.001 ng/µl +DWV	1.4 ng/µl	1.4 ng/µl +PBS	1.4 ng/µl +DWV
	Normal wing	control													
		PBS	0.962												
		0.001 ng/µl	1.000	1.000											
		0.001 ng/µl+ PBS	1.000	1.000	1.000										
		1.4 ng/µl	0.220	0.774	0.663	0.783									
		1.4 ng/µl+ PBS	0.753	0.936	0.888	0.906	1.000								
p-value	Deformed wing	DWV	0.022	0.022	0.022	0.022	0.022	0.022							
		0.001 ng/µl	0.103	0.664	0.572	0.737	1.000	1.000	0.022						
		0.001 ng/µl +PBS	0.396	0.654	0.572	0.62	0.991	1.000	0.022	0.978					
		0.001ng/µl +DWV	0.014	0.014	0.014	0.014	0.015	0.014	1.000	0.015	0.014				
		1.4 ng/µl	0.031	0.079	0.081	0.167	0.583	0.998	0.022	0.378	1.000	0.015			
		1.4 ng/µl +PBS	0.394	0.531	0.473	0.488	0.854	0.995	0.022	0.822	1.000	0.014	1.000		
		1.4 ng/µl +DWV	0.010	0.010	0.010	0.010	0.010	0.010	1.000	0.010	0.010	1.000	0.011	0.010	

Table S5 Statistic of *defensin* in newly emerged adult honey bees

Games-Howell Post-Hoc Test												
	**C**	**PBS**	**DWV**	**0.001+n**	**0.001+d**	**0.001+PBS+n**	**0.001+PBS+d**	**0.001+DWV+d**	**1.4+n**	**1.4+d**	**1.4+PBS+n**	**1.4+PBS+d**
**PBS**	1.000											
**DWV**	0.003	0.088										
**0.001+n**	1.000	1.000	0.133									
**0.001+d**	0.029	0.432	0.026	0.557								
**0.001+PBS+n**	0.320	0.981	0.002	0.981	0.003							
**0.001+PBS+d**	0.960	0.980	0.316	0.993	0.985	0.685						
**0.001+DWV+d**	0.951	0.979	0.258	0.992	0.966	0.639	0.992					
**1.4+n**	0.066	0.470	0.048	0.596	0.997	0.012	0.596	0.982				
**1.4+d**	1.000	1.000	0.655	1.000	0.998	0.986	1.000	1.000	0.999			
**1.4+PBS+n**	0.651	0.849	0.109	0.923	0.943	0.290	0.923	1.000	0.972	1.000		
**1.4+PBS+d**	1.000	1.000	0.078	1.000	0.485	0.815	1.000	0.998	0.530	1.000	0.949	
**1.4+DWV+d**	0.179	0.455	0.102	0.587	1.000	0.083	0.587	0.979	1.000	0.998	0.978	0.537

n = normal wing
d = deformed wing

Table S6 Statistic of *abaecin* in newly emerged adult honey bees

Games-Howell Post-Hoc Test												
	**C**	**PBS**	**DWV**	**0.001+n**	**0.001+d**	**0.001+PBS+n**	**0.001+PBS+d**	**0.001+DWV+d**	**1.4+n**	**1.4+d**	**1.4+PBS+n**	**1.4+PBS+d**
**PBS**	0.886											
**DWV**	0.034	0.372										
**0.001+n**	0.874	1.000	0.103									
**0.001+d**	0.992	0.771	0.125	0.777								
**0.001+PBS+n**	0.999	0.940	0.056	0.940	0.932							
**0.001+PBS+d**	1.000	0.967	0.330	0.981	1.000	1.000						
**0.001+DWV+d**	0.181	0.990	0.315	0.732	0.374	0.200	0.722					
**1.4+n**	0.710	1.000	0.711	0.983	0.604	0.777	0.872	1.000				
**1.4+d**	0.434	0.254	0.059	0.258	0.866	0.367	0.853	0.143	0.203			
**1.4+PBS+n**	0.667	1.000	0.080	1.000	0.711	0.684	0.968	0.536	0.972	0.261		
**1.4+PBS+d**	1.000	0.965	0.078	0.981	0.992	1.000	1.000	0.414	0.819	0.439	0.935	
**1.4+DWV+d**	0.660	1.000	0.454	0.993	0.589	0.736	0.887	1.000	1.000	0.183	0.983	0.815

n = normal wing
d = deformed wing

Table S7 Statistic of *hymenoptaecin* in newly emerged adult honey bees

Games-Howell Post-Hoc Test												
	**C**	**PBS**	**DWV**	**0.001+n**	**0.001+d**	**0.001+PBS+n**	**0.001+PBS+d**	**0.001+DWV+d**	**1.4+n**	**1.4+d**	**1.4+PBS+n**	**1.4+PBS+d**
**PBS**	0.388											
**DWV**	0.011	0.032										
**0.001+n**	0.429	0.999	0.161									
**0.001+d**	0.250	0.700	0.507	0.967								
**0.001+PBS+n**	0.989	0.554	0.008	0.533	0.289							
**0.001+PBS+d**	0.349	0.736	0.826	0.945	1.000	0.394						
**0.001+DWV+d**	0.005	0.116	0.550	0.338	0.837	0.012	0.993					
**1.4+n**	0.517	0.929	0.738	0.997	1.000	0.582	1.000	0.949				
**1.4+d**	0.045	0.223	0.316	0.677	1.000	0.045	1.000	0.742	1.000			
**1.4+PBS+n**	0.004	0.390	0.103	0.916	1.000	0.017	0.999	0.011	1.000	0.815		
**1.4+PBS+d**	0.325	1.000	0.064	1.000	0.877	0.434	0.866	0.185	0.984	0.411	0.665	
**1.4+DWV+d**	0.006	0.042	0.999	0.210	0.625	0.005	0.913	0.736	0.826	0.443	0.093	0.086

n = normal wing
d = deformed wing

Table S8 Statistic of *caspase3-like* in newly emerged adult honey bees

Games-Howell Post-Hoc Test												
	**C**	**PBS**	**DWV**	**0.001+n**	**0.001+d**	**0.001+PBS+n**	**0.001+PBS+d**	**0.001+DWV+d**	**1.4+n**	**1.4+d**	**1.4+PBS+n**	**1.4+PBS+d**
**PBS**	0.905											
**DWV**	0.355	0.193										
**0.001+n**	0.585	1.000	0.022									
**0.001+d**	0.246	0.826	0.001	0.603								
**0.001+PBS+n**	1.000	0.998	0.919	0.984	0.853							
**0.001+PBS+d**	0.488	0.879	0.208	0.882	0.997	0.819						
**0.001+DWV+d**	0.774	0.392	1.000	0.291	0.198	0.973	0.219					
**1.4+n**	0.629	1.000	0.052	1.000	0.773	0.985	0.903	0.287				
**1.4+d**	0.664	1.000	0.047	1.000	0.673	0.989	0.877	0.302	1.000			
**1.4+PBS+n**	0.808	1.000	0.025	0.996	0.277	0.998	0.761	0.366	0.997	1.000		
**1.4+PBS+d**	1.000	0.999	0.547	0.970	0.684	1.000	0.727	0.771	0.974	0.983	0.999	
**1.4+DWV+d**	0.610	1.000	0.008	1.000	0.330	0.989	0.838	0.312	1.000	1.000	0.999	0.980

n = normal wing
d = deformed wing

Table S9 Statistic of *caspase8-like* in newly emerged adult honey bees

Games-Howell Post-Hoc Test												
	**C**	**PBS**	**DWV**	**0.001+n**	**0.001+d**	**0.001+PBS+n**	**0.001+PBS+d**	**0.001+DWV+d**	**1.4+n**	**1.4+d**	**1.4+PBS+n**	**1.4+PBS+d**
**PBS**	0.993											
**DWV**	0.071	0.070										
**0.001+n**	0.392	0.847	0.014									
**0.001+d**	0.441	0.880	0.019	1.000								
**0.001+PBS+n**	1.000	0.929	0.033	0.228	0.279							
**0.001+PBS+d**	0.161	0.466	0.006	0.983	0.983	0.075						
**0.001+DWV+d**	1.000	1.000	0.046	0.528	0.585	0.994	0.211					
**1.4+n**	0.768	1.000	0.016	0.745	0.809	0.266	0.277	0.939				
**1.4+d**	0.140	0.361	0.011	0.875	0.880	0.104	1.000	0.182	0.282			
**1.4+PBS+n**	1.000	0.999	0.022	0.378	0.446	0.962	0.123	1.000	0.689	0.151		
**1.4+PBS+d**	0.991	1.000	0.317	1.000	1.000	0.970	0.967	0.999	1.000	0.907	0.998	
**1.4+DWV+d**	0.514	0.916	0.031	1.000	1.000	0.358	0.985	0.663	0.878	0.896	0.540	1.000

n = normal wing
d = deformed wing

Table S10 Statistic of *caspase9-like* in newly emerged adult honey bees

Games-Howell Post-Hoc Test												
	**C**	**PBS**	**DWV**	**0.001+n**	**0.001+d**	**0.001+PBS+n**	**0.001+PBS+d**	**0.001+DWV+d**	**1.4+n**	**1.4+d**	**1.4+PBS+n**	**1.4+PBS+d**
**PBS**	1.000											
**DWV**	0.018	0.115										
**0.001+n**	0.999	1.000	0.036									
**0.001+d**	0.283	0.794	0.075	0.115								
**0.001+PBS+n**	1.000	1.000	0.026	1.000	0.187							
**0.001+PBS+d**	0.987	1.000	0.053	1.000	0.105	1.000						
**0.001+DWV+d**	0.864	0.999	0.014	0.392	0.052	0.552	0.308					
**1.4+n**	0.999	1.000	0.014	0.864	0.157	0.956	0.724	0.997				
**1.4+d**	1.000	1.000	0.098	1.000	0.901	1.000	1.000	0.913	0.993			
**1.4+PBS+n**	0.999	1.000	0.014	0.865	0.169	0.954	0.732	0.998	1.000	0.993		
**1.4+PBS+d**	0.600	0.825	0.047	0.496	0.268	0.524	0.470	0.851	0.728	0.612	0.738	
**1.4+DWV+d**	0.998	1.000	0.026	0.632	0.031	0.872	0.358	0.961	1.000	0.990	1.000	0.683

n = normal wing
d = deformed wing

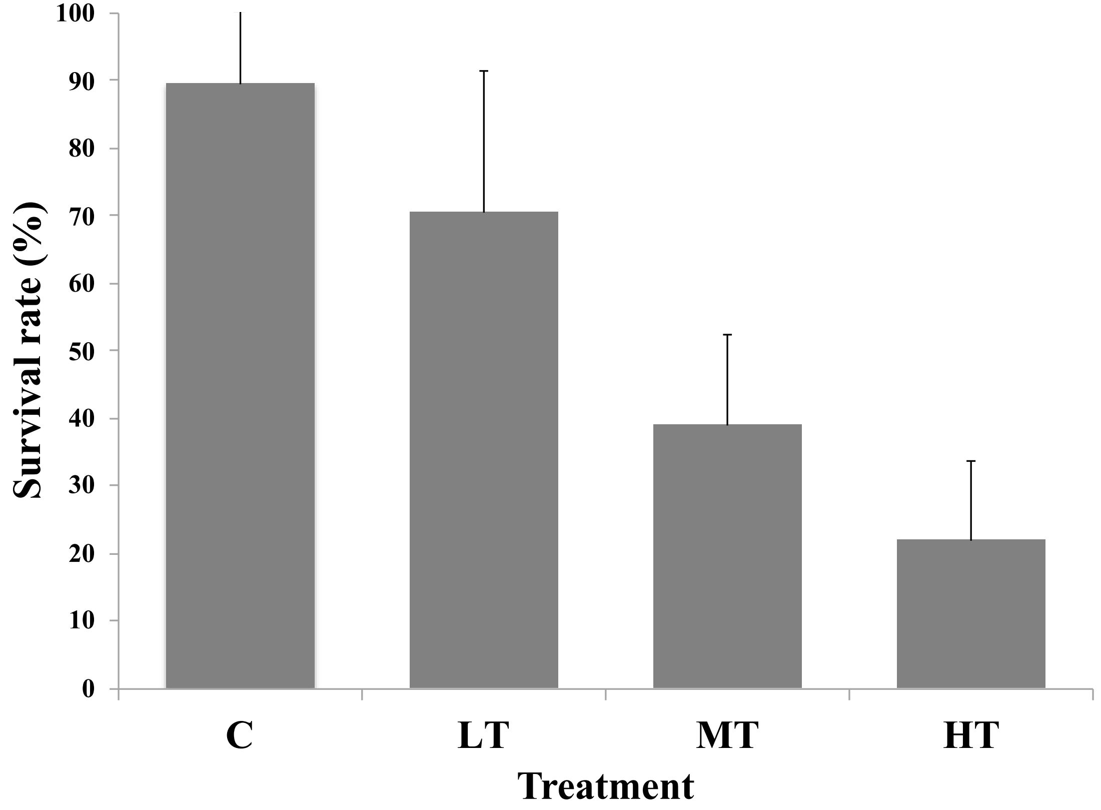

Figure S1. The percentage of survival rate of white-eyed pupae honey bees at 12 days post-fed.

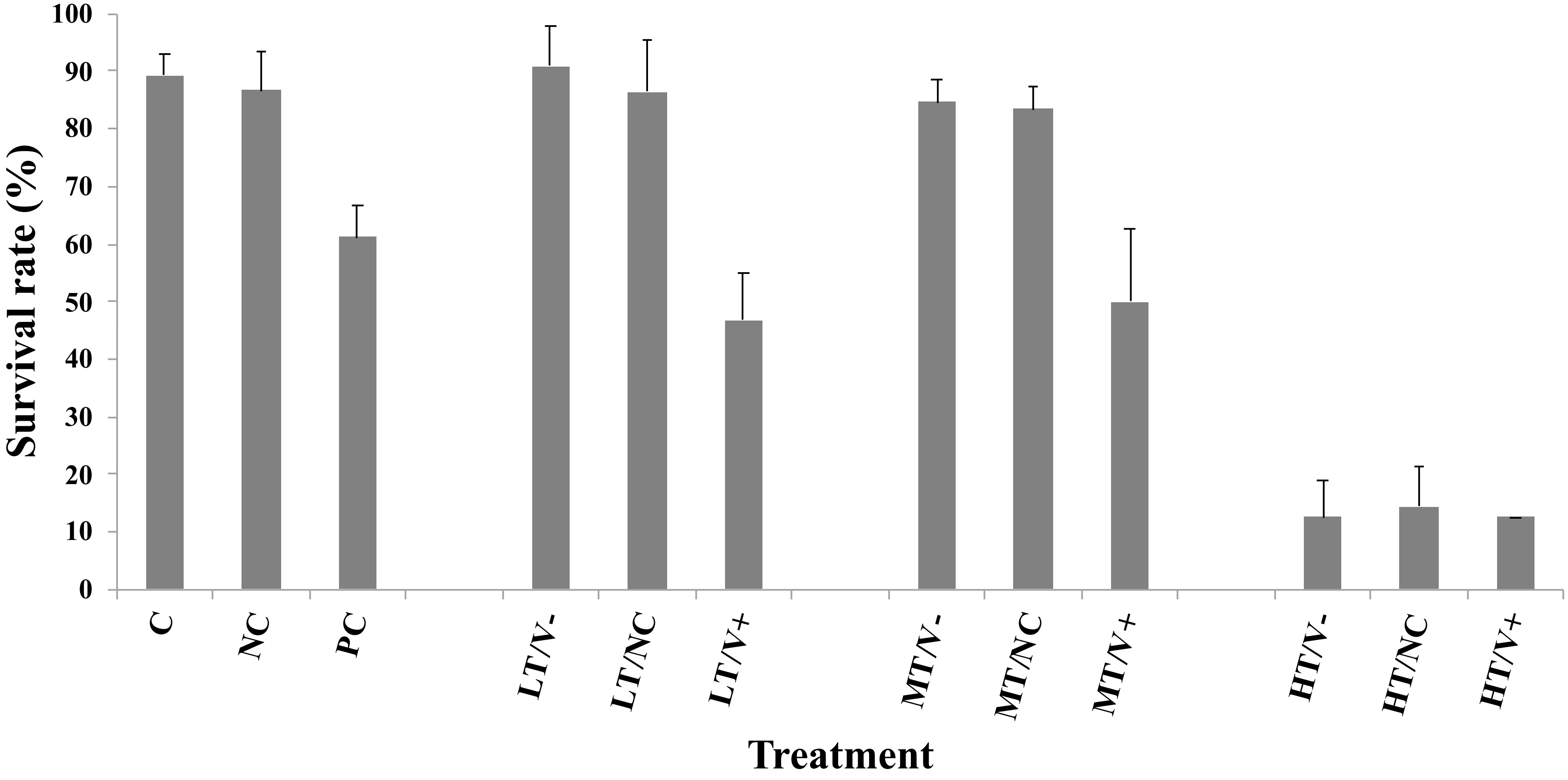

Figure S2. The percentage of survival rates in newly emerged adult honey bees.
